# Laboratory Investigation of <i>Salmonella enterica</i> serovar Poona Outbreak in California: Comparison of Pulsed-Field Gel Electrophoresis (PFGE) and Whole Genome Sequencing (WGS) Results

**DOI:** 10.1371/currents.outbreaks.1bb3e36e74bd5779bc43ac3a8dae52e6

**Published:** 2016-11-22

**Authors:** Varvara K. Kozyreva, John Crandall, Ashley Sabol, Alyssa Poe, Peng Zhang, Jeniffer Concepción-Acevedo, Morgan N. Schroeder, Darlene Wagner, Jeffrey Higa, Eija Trees, Vishnu Chaturvedi

**Affiliations:** California Department of Public Health, Richmond, CA, USA; California Department of Public Health, Richmond, CA, USA; Centers for Disease Control and Prevention, Atlanta, GA, USA; California Department of Public Health, Richmond, CA, USA; California Department of Public Health, Richmond, CA, USA; Centers for Disease Control and Prevention, Atlanta, GA, USA; Centers for Disease Control and Prevention, Atlanta, GA, USA; Centers for Disease Control and Prevention, Atlanta, GA, USA; California Department of Public Health, Los Angeles, CA, USA; Centers for Disease Control and Prevention, Atlanta, GA, USA; California Department of Public Health, Richmond, CA, USA

**Keywords:** Outbreak, phylogenetic analysis, pulsed-field gel electrophoresis, Salmonella serovar Poona, whole-genome sequencing

## Abstract

**Introduction:**

Recently, *Salmonella enterica *serovar Poona caused a multistate outbreak, with 245 out of 907 cases occurring in California. We report a comparison of pulsed-field gel electrophoresis (PFGE) results with whole genome sequencing (WGS) for genotyping of *Salmonella* Poona isolates.

**Methods:**

CA *Salmonella* Poona isolates, collected from July to August 2015, were genotyped by PFGE using XbaI restriction enzyme. WGS was done using Nextera XT library kit with 2x300 bp or 2x250 bp sequencing chemistry on the Illumina MiSeq Sequencer.  Reads were mapped to the de novo assembled serovar Poona draft genome (48 contigs, N50= 223,917) from the outbreak using CLCbio GW 8.0.2. The phylogenetic tree was generated based on hqSNPs calling. Genomes were annotated with CGE and PHAST online tools. In silico MLST was performed using the CGE online tool.

**Results:**

Human (14) and cucumber (2) *Salmonella* Poona isolates exhibited 3 possibly related PFGE patterns (JL6X01.0018 [predominant], JL6X01.0375, JL6X01.0778).  All isolates that were related by PFGE also clustered together according to the WGS. One isolate with a divergent PFGE pattern (JL6X01.0776) served as an outlier in the phylogenetic analysis and substantially differed from the outbreak clade by WGS. All outbreak isolates were assigned to MLST sequence type 447. The majority of the outbreak-related isolates possessed the same set of *Salmonella* Pathogenicity Islands with few variations. One outbreak isolate was sequenced and analyzed independently by CDC and CDPH laboratories; there was 0 SNP difference in results. Additional two isolates were sequenced by CDC and the raw data was processed through CDPH and CDC analysis pipelines. Both data analysis pipelines also generated concordant results.

**Discussion:**

PFGE and WGS results for the recent CA *Salmonella enterica *serovar Poona outbreak provided concordant assignment of the isolates to the outbreak cluster. WGS allowed more robust determination of genetic relatedness, provided information regarding MLST-type, pathogenicity genes, and bacteriophage content. WGS data obtained independently at two laboratories showed complete agreement.

## The Study

*Salmonella enterica* serotype Poona is a relatively rare serotype in the United States, responsible for about 1% of reported human *Salmonella* cases^1^. *Salmonella *Poona outbreaks are commonly attributed to farm-produced crops and goods^2^. Serotype Poona has also caused outbreaks associated with pet reptile exposure^3^. Recently, *Salmonella* Poona caused a multistate outbreak, with 245 out of 907 cases occurring in California. A multistate investigation identified imported cucumbers distributed by Company A as the likely source of the outbreak^4^. We report results of a real-time comparison of pulsed-field gel electrophoresis (PFGE) and whole genome sequencing (WGS) for this *Salmonella* Poona outbreak investigation.

The *Salmonella* Poona isolates from California analyzed in this study are listed in Table 1. At the California Department of Public Health (CDPH), PFGE was performed using *Xba*I macrorestriction per PulseNet USA protocol^5^. For WGS, DNA libraries were prepared using Nextera XT kit (Illumina Inc., San Diego, CA) and sequenced with 2x300 bp or 2x250 bp chemistry on the Illumina MiSeq at 49-124x sequencing coverage (see NCBI accession numbers for the corresponding sequences in Table 1). Time-to-results was 4 days for PFGE and 7 days for WGS. The analysis of sequencing data at CDPH was performed as follows:

Genomes were annotated with prokka v1.1 tool^6^, the Center for Genomic Epidemiology (CGE), and Phage Search Tool (PHAST) online resources^7^^,^^8^. *In silico* multi-locus sequence typing (MLST) was performed using the CGE online tool^9^ against the 7-gene MLST database of University of Warwick (http://mlst.warwick.ac.uk/mlst/dbs/Senterica). The *Salmonella* Pathogenicity Islands (SPI) were characterized using SPIFinder 1.0 CGE tool (https://cge.cbs.dtu.dk/services/SPIFinder/).

Paired-end reads were mapped to the *de novo* assembled outbreak *Salmonella* Poona M15X04725 draft genome (48 contigs, N50= 223,917) using CLCbio Genomic Workbench 8.0.2 (Qiagen, Aarhus, Denmark). Genes of mobile and prophage elements in the reference *Salmonella* Poona M15X04725 genome were annotated using prokka v1.1 and PHAST tools and consequently masked from mapping in CLCbio Genomic Workbench 8.0.2. SNPs were then called in coding and non-coding genome areas using SAMtools mpileup (v.1.2;^10^) and converted into VCF matrix using bcftools (v0.1.19; http://samtools.github.io/bcftools/). Variants were parsed using vcftools (v.0.1.12b;^11^) to include only high-quality SNPs (hqSNPs) with minimum position coverage ≥30x and minimum quality of the base > 200 (--min-meanDP 30; --minQ 200), with InDels and the heterozygote calls excluded. The Maximum Likelihood phylogenetic tree was generated based on hqSNPs calls under the Jukes-Cantor nucleotide substitution model; with 100 bootstrap replicates.

At the US Centers for Disease Control and Prevention (CDC) LyveSET 1.1.4 SNP calling pipeline (http://www.github.com/lskatz/lyve-SET) was applied with reads trimmed using fastx_trimmer 5 bases from 5’ ends before mapping single-end by SMALT. SNPs were called using Varscan at > 20x coverage, > 95% read support, and ≥5 bp apart. The draft assembly of FDA00009409 from the outbreak (38 contigs) was used as a reference without prophage masking.

Fourteen human and two cucumber *Salmonella* Poona isolates analyzed in this study exhibited 3 possibly related PFGE patterns (JL6X01.0018 [predominant], JL6X01.0375, JL6X01.0778). The isolates were selected to include representatives of the three above mentioned PFGE patterns and epidemiologically representative isolates with and without links to the cucumber source. In the interest of the cost effectiveness, the WGS was limited to 16 isolates since the detected PFGE patterns were rare in California and only few SNP differences were found among the sampled isolates. Of the 14 case-patients whose isolates were analyzed, 10 (71%) reported consuming cucumbers during the week preceding their illness onsets, 2 case-patients denied consuming any cucumbers, and cucumber exposure was unknown for 2 case-patients (Table 1. The cucumber exposures reported by the 10 case-patients were all linked to Company A. One isolate (M15X03586) with a divergent PFGE pattern (JL6X01.0776) served as an outlier in the phylogenetic analysis (Table 1, Figure 1A). Cucumber exposure for the patient was unknown; the patient was not considered part of the outbreak cluster based on PFGE.

**Phylogeny of Salmonella enterica serovar Poona based on single nucleotide polymorphism (SNP) differences discovered between the isolates by whole genome sequencing (WGS) vs. phylogeny based on PFGE profiles clustering.**
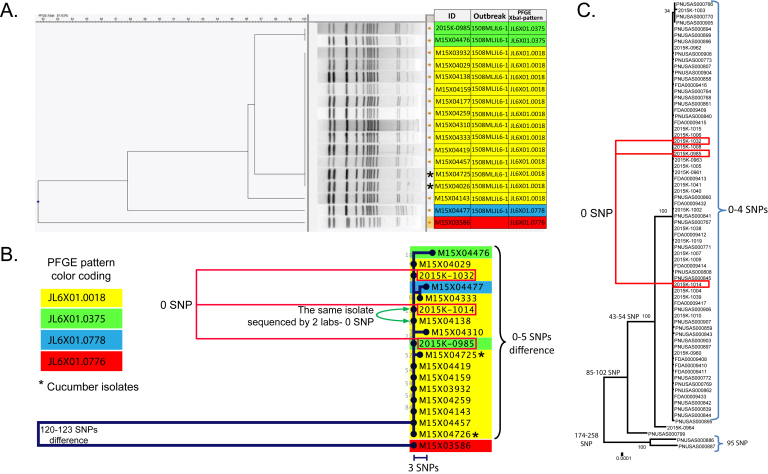
A- Pulsed-field gel electrophoresis (PFGE) dendrogram of the California isolates; B- Maximum likelihood tree generated by California Department of Public Health (CDPH) for California outbreak isolates. Background coloring designates PFGE pattern. Red boxes designate sequences generated by CDC; C- Maximum likelihood tree generated by Centers for Disease Control and Prevention (CDC) for multistate investigation, including California. Red boxes designate selected isolates from California.

**Pairwise comparison of single nucleotide polymorphism (SNP) differences between Salmonella Poona isolates from California.**
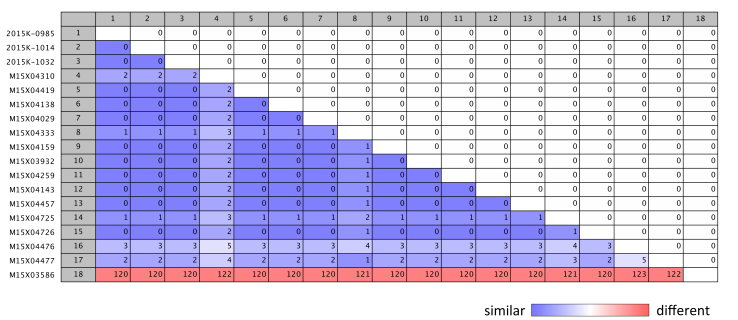

**Figure:**
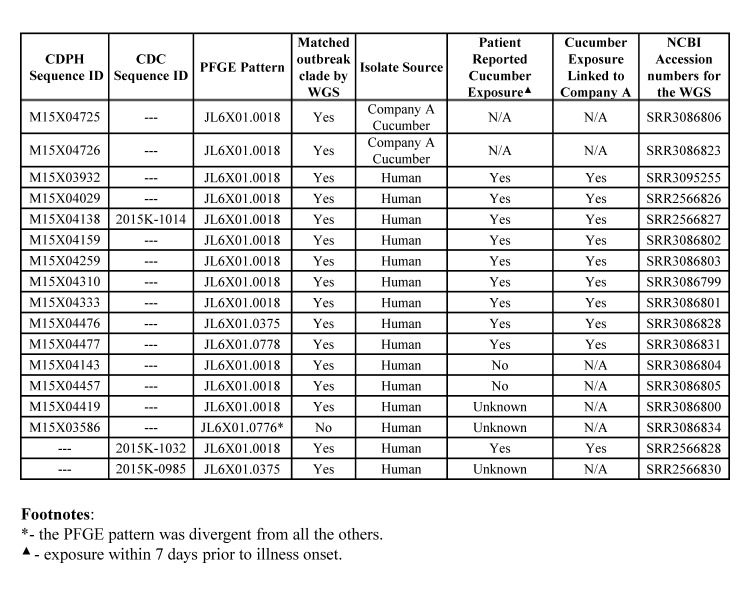
**Table 1.***Salmonella enterica* serotype Poona isolates, California (N=17): pulsed-field gel electrophoresis (PFGE) profiles, whole genome sequencing (WGS)-based clustering, and patient-reported cucumber exposure.

All 16 isolates that were highly similar by PFGE also clustered together according to WGS. WGS of cucumber isolates showed close relatedness to the genomes of human isolates, with only 0-3 SNPs difference (Figure 1B, 2). The isolate with the divergent PFGE pattern JL6X01.0776 was substantially different (120-123 hqSNPs) from the outbreak clade by WGS (Figure 1A vs. 1B). One outbreak isolate M15X04138 was sequenced and analyzed independently by CDC and CDPH laboratories; no SNP differences in sequences were detected. Two additional isolates were sequenced by CDC, and the raw data was processed through CDPH and CDC analysis pipelines. Both data analysis pipelines generated the same results (Figure 1B vs. 1C).

All outbreak isolates were assigned to the MLST sequence type 447 (ST447). The divergent outgroup strain had a single mutation in *hemD* gene locus, which did not correspond to any known allele in the MLST database. There was only one entry of ST447 in MLST database; *Salmonella *strain 3854/83 isolated in 1982 from a wild rodent in India.

The majority of the outbreak-related isolates possessed the same set of *Salmonella* Pathogenicity Islands (SPI), with variability mostly caused by the absence of SPI-2 or SPI-3, which encode Type III secretion system and invasion functions, respectively (Table 2).

**Figure:**
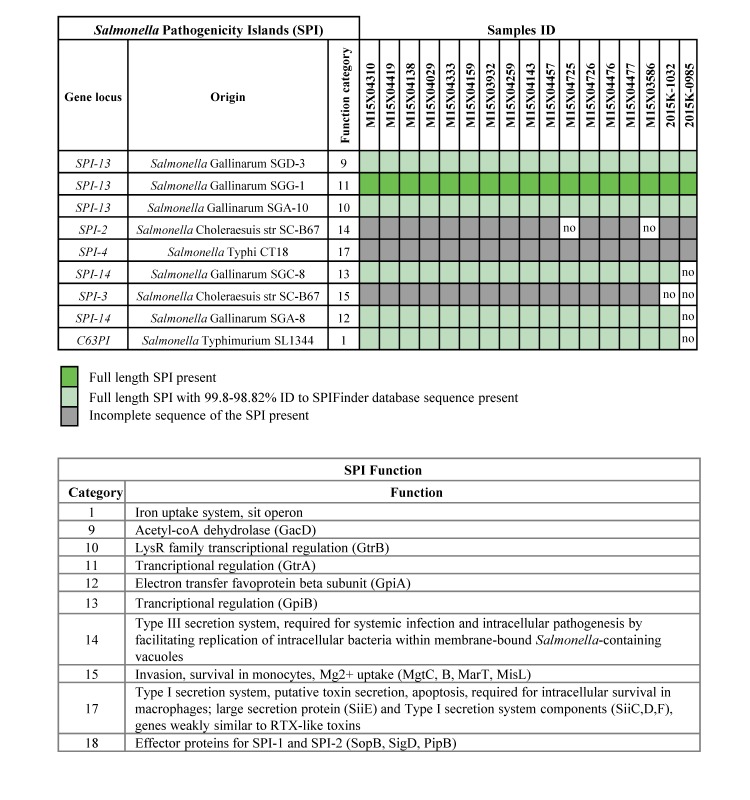
**Table 2.***Salmonella* Pathogenicity Islands in outbreak-related *Salmonella* Poona isolates.

All isolates possessed phage elements homologous to *Salmonella* phage Fels-1 (NC_010391) and partial *Enterobacteria* phage P4 (NC_001609). Four isolates harbored the genes homologous to an additional bacteriophage of either of the following types: *Synechococcus* phage S-SKS1 (NC_020851), *Ectocarpus siliculosus* virus 1 (NC_002687), *Enterobacteria* phage ES18 (NC_006949), or *Enterobacteria* phage P1 (NC_005856). Interestingly, phage genes found in one of the isolates were homologous to S-SKS1 bacteriophage, which was described as a virus infecting *Synechococcus*, a genus of Cyanobacteria known to be one of the predominant prokaryotic components of the oceans’ picophytoplankton^12^. Another isolate possessed genes homologous to EsV-1 bacteriophage, characterized previously as a bacteriophage of the marine filamentous brown algae *Ectocarpus siliculosus*^13^. The sequences identified as incomplete S-SKS1 or EsV-1 phages were integrated into the larger contigs with clear *Salmonella* identity according to BLAST search. The marine phage-like sequences were also common in other *Salmonella enterica* serotypes found in NCBI database and seem to represent a part of the *Salmonella* accessory genome.

WGS has proven to be a powerful tool for the investigation of outbreaks caused by *Salmonella enterica*, providing great epidemiological concordance and higher resolution than PFGE, the traditional method for *Salmonella* strain subtyping^14^^,^^15^^,^^16^. In our study, WGS confirmed the genetic relatedness of strains with similar PFGE patterns. Outbreak-related isolates exhibited 3 PFGE patterns which complicated assignment of the isolates to a single outbreak source. Particularly, isolate M15X04477 with the pattern JL6X01.0778 differed by 3 bands from the predominant outbreak PFGE-type, thus introducing a higher degree of uncertainty regarding the inclusion of this isolate in the outbreak cluster even though the epidemiologic information supported a possible link to the outbreak. WGS, on the other hand, showed unambiguous clustering of all epidemiologically-related isolates together and clarified genetic relatedness of M15X04477 to the other outbreak isolates. Two case-patients denying cucumber consumption also clustered with the outbreak, which could be explained by possible secondary transmission, cross-contamination of other foods, poor food history recall, or case-patients’ not noticing cucumbers as part of a multi-ingredient meal such as a salad or a sandwich. In support of the high reproducibility of the method, the results of WGS performed by two different laboratories were identical. Additionally, WGS provided information about the MLST sequence type and acquired components of the genome, allowing for more comprehensive characterization of the isolates. Though MLST usually provides poor resolution between *Salmonella* strains, it is helpful for understanding the global epidemiology of infectious clones^17^. Diversity in accessory genes of the studied isolates demonstrates the plasticity of the *Salmonella* Poona genome which is potentially responsible for variations in PFGE patterns that do not necessarily reflect the phylogenetic distance between the isolates^18^. Since the loss/acquisition of mobile elements does not affect genome-wide SNPs calling, it explains higher evolutionary congruence of WGS-inferred phylogeny compared with PFGE. Noteworthy, WGS results were acquired in 7 working days from the time of pure culture reception by the laboratory. Our experience suggests that WGS can be used for routine epidemiological subtyping.

## Conclusions

Real-time PFGE and WGS genotyping results from this *Salmonella* Poona outbreak provided concordant assignment of isolates to the outbreak cluster by both methods. However, the WGS allowed for a more unequivocal determination of the genetic relatedness of the isolates than PFGE. WGS provided additional information regarding MLST type, pathogenicity genes, and bacteriophage content of the isolates. WGS data obtained independently at two laboratories showed complete agreement.

## Data Availability Statement

The raw reads for sequenced here samples were submitted to NCBI SRA archive under accession numbers SRR2566826- SRR2566828, SRR2566830, SRR3086799- SRR3086806, SRR3086823, SRR3086828, SRR3086831, SRR3086834, SRR3095255 (see Table 1 for details).

## Competing Interest Statement

The authors have declared that no competing interests exist.

## Corresponding Authors

Vishnu Chaturvedi: Vishnu.Chaturvedi@cdph.ca.gov

Varvara K. Kozyreva: Varvara.Kozyreva@cdph.ca.gov
